# Role of COVID-19 infection status on the prediction of future infection: Immunity or susceptibility

**DOI:** 10.1371/journal.pone.0317959

**Published:** 2025-03-26

**Authors:** Fateme Nikbakht, Hamid Heidarian Miri, Ehsan Mosafarkhani, Fatemeh Sharifjafari, Ali Taghipour

**Affiliations:** 1 Department of Epidemiology, School of Health, Mashhad University of Medical Sciences, Mashhad, Iran; 2 School of Nursing and Midwifery, University College Cork, Cork, Ireland; 3 Management and Social Determinants of Health Research Center, Mashhad University of Medical Sciences, Mashhad, Iran; 4 Department of Medicine, The University of Georgia, Tbilisi, Georgia; 5 Social Determinants of Health Research Center, Mashhad University of Medical Sciences, Mashhad, Iran.; Gabriele d'Annunzio University of Chieti and Pescara: Universita degli Studi Gabriele d'Annunzio Chieti Pescara, ITALY

## Abstract

**Background:**

COVID-19 has rapidly spread around the world, and the duration of protective immunity against the virus remains unknown. Evidence suggests that patients with a confirmed COVID-19 infection may experience reinfection. The aim of this study is to determine the relationship between COVID-19 reinfection and previous infection history in the population covered by Mashhad University of Medical Sciences.

**Methods:**

This population-based, historical cohort study included all individuals with health records at the health service centers of Mashhad University of Medical Sciences who underwent PCR testing during the study period (April 1, 2020, up to February 19, 2022). The data were analyzed by calculating the infection rate in both PCR-positive and negative individuals, and estimating the adjusted rate ratio using Poisson regression.

**Results:**

The results of this study in the entire population showed that the incidence rate in people with a history of primary COVID-19 infection was 13% higher than that in people who had no history of this disease. However, in the group that received the vaccine prior to the first PCR test, the incidence rate was lower among individuals with a positive first test result (IRR =  0.71) compared to those with a negative first test result.

**Conclusion:**

The study reveals that prior COVID-19 infection does not ensure immunity and may increase the risk of reinfection, particularly among men and younger individuals. Vaccination appears to complicate this dynamic, as those with multiple vaccine doses showed higher reinfection rates compared to those with fewer doses. These findings highlight the need for ongoing research and tailored public health strategies to address the complexities of COVID-19 immunity and reinfection.

## Introduction

More than three years after the onset of the COVID-19 pandemic, reinfection remains a topic of debate. The disease spread rapidly throughout most countries worldwide [1]. However, despite its rapid spread, the duration and extent of protective immunity against the virus remain unknown [2]. This virus is genetically 70% similar to SARS [3]. However, despite these genetic similarities, the presence of specific viral proteins in the structure of the Corona virus has made it much more destructive [4]. Given the disease’s specific characteristics and pathogenicity, there is a concern about the possibility of reinfection. Reinfection could be defined as an individual who was infected, recovered, and then later became infected again [5]. Also, a suspected case of COVID-19 reinfection is defined as two positive PCR tests with an interval of more than 90 days and a minimum interval of seven days without symptoms between the tests [6].

Although exposure to some viral infections can provide permanent immunity, other viruses can cause reinfection in individuals [2]. Studies show that immunity can develop after a single COVID-19 infection. However, this immunity is short-lived, which explains the possibility of reinfection [7]. Risk factors for the reactivation of COVID-19 are related to the type of immunosuppressive treatments and host-related factors, including old age, gender, and comorbid diseases such as diabetes, heart disease, obesity, and cancer [8].

To significantly reduce morbidity and mortality caused by COVID-19, an effective and safe vaccine must be made available to the public rapidly and on a wide scale as soon as it is developed. However, the availability of a vaccine alone is not sufficient to guarantee widespread immunological protection [9]. Factors known to affect vaccination intention include socio-demographic individual characteristics, beliefs, and personal experiences, as well as external or larger organizational factors [10]. Vaccination hesitancy is a major barrier to vaccine uptake and the achievement of herd immunity needed to protect the most vulnerable populations [9].

The purpose of this study is to investigate the relationship between COVID-19 reinfection and previous infection history among individuals covered by Mashhad University of Medical Sciences. By addressing the existing knowledge gap regarding reinfection rates and the limitations of previous research, this study aims to clarify how protective immunity from initial infections or vaccinations impacts virus transmission and disease severity. The findings will provide valuable insights into demographic factors, such as age, gender, and comorbidities, that influence reinfection rates.

## Materials and methods

### Study design

This population-based, historical cohort study, which focused on the Iranian population, used multiple databases that recorded cases of patients with COVID-19. The study extracted and analyzed information related to the PCR test in all age groups from the beginning of the PCR test at Mashhad University of Medical Sciences (April 1, 2020) up to February 19, 2022, from these systems.

### Study setting and participants

Individuals were eligible to participate in this study if they had undergone at least one PCR test. Furthermore, individuals who were tested at health centers affiliated with Mashhad University of Medical Sciences but did not fall within the population covered by the university were excluded from the study, regardless of the reason for their testing. In this study, COVID-19 reinfection was considered an outcome, while COVID-19 infection was defined the exposure. COVID-19 reinfection was defined as the presence of two reported positive tests separated by more than 90 days. Other variables considered included gender, age groups, place of residence, comorbidities, vaccination status, and type of vaccine received. These were classified as independent and confounding variables.

### Data sources and measurement

This study that utilized information from three sources: the Sina Electronic Health Registration System database, the Medical Care Monitoring Center (MCMC), and the Hospital Information System (HIS). The information pertaining to the PCR test in all age groups was gathered from these systems, starting from the initiation of the PCR test at Mashhad University of Medical Sciences on April 1, 2020, until February 19, 2022 (data accessed February 20, 2022). Since 2016, the Sina Electronic Health Registration System database (SinaEHR) was utilized to electronically register the health records of clients in the health centers of Razavi Khorasan province. This integrated system encompasses crucial data such as demographic information, individual health records, laboratory results, screening forms, and specialized care for various age groups. Moreover, in response to COVID-19, the system also contains records of PCR tests conducted at health centers and information about COVID-19 vaccinations. These databases are widely used in various epidemiological and health policy studies due to their integrity and completeness.

### Statistical analysis

In this study, we investigated all positive and negative PCR tests in all individuals with health records at the health service centers of Mashhad University of Medical Sciences. Firstly, we conducted a descriptive analysis of the data. Next, we calculated and compared the infection rates among individuals with positive and negative tests. The infection rate was determined by dividing the number of COVID-19 reinfections by the cumulative number of person-days at risk. Finally, we adjusted the rate ratio and 95% confidence interval for sex and age groups using the infection rates in PCR positive and negative individuals. Also, protection against COVID-19 reinfection was calculated as 1 minus the adjusted rate ratio. In this study, Poisson regression was used to check the relationship. Variables that had a P-value less than 0.2 in the univariate analysis were entered into the multivariable analysis. Also to ensure the robustness of our regression analyses, we conducted a thorough examination of collinearity among the variables included in our models. In our analysis, we computed VIFs for all predictors included in the regression model. If any predictors exhibited high VIF values (greater than 5), we considered potential strategies to mitigate multicollinearity, such as removing highly correlated variables or combining them into a single predictor. Data were analyzed with Stata version 14. The analysis was conducted on five classified groups to consider the timing of vaccination in relation to exposure and outcome. Further details about the analysis can be found in S1–S4 Tables.

### Ethical statement

The Ethics Committee of Mashhad University of Medical Sciences waived the need for individual informed consent because of the study’s retrospective nature and the use of de-identified data. However considering that in this study, the researchers needed to merge data from three sources, they obtained permission from Mashhad University of Medical Sciences and adhered to the ethics code to access the nationality code of the individuals only for research purposes (Ethics Code:IR.MUMS.FHMPM.REC.1400.081). This allowed them to use the information effectively.

## Results

Among 846,318 people, 52.53% were men. The mean age of the study Participants was 37.85 ± 16.65 years. In terms of place of residence, 82.28% of the people lived in cities, and the residents of urban areas had experienced COVID-19 more than the residents of rural areas. In the whole population, 42.59% of the people had received two doses of the vaccine. Only 8.71% of the research population had a history of the diseases examined in this study. The categories of heart diseases, respiratory, kidney, diabetes and blood pressure are considered in this study. The demographic characteristics of the study population are detailed in Table 1 according to the primary COVID-19 infection status.

**Table 1:** Demographic characteristics of the study population by primary COVID-19 infection status.

Variable		Without primary infection	With primary infection	P-value^*^
		Frequency (percentage)	Frequency (percentage)	
Gender	Female	160977 (48.20)	240787 (47.00)	<0.001
	Male	172977 (51.80)	271577 (53.00)	
Age group	0–59 years	303016 (90.74)	447629 (87.37)	<0.001
	≥60 years	30938 (9.26)	64735 (12.63)	
Place of residence	Rural	89918 (26.93)	60008 (11.71)	<0.001
	City	244036 (73.07)	452356 (88.29)	
Comorbidities	Without	304656 (91.23)	467936 (91.33)	0.104
	With	29298 (8.77)	44428 (8.67)	
Vaccination status	Unvaccinated	53559 (16.04)	52921 (10.33)	<0.001
	First dose	33101 (9.91)	47814 (9.33)	
	Second doses	141204 (42.28)	219237 (42.79)	
	Three or more doses	106090 (31.77)	192392 (37.55)	
Vaccine type	Unvaccinated	53559 (16.04)	52921 (10.33)	<0.001
	Inactivated	256127 (76.70)	402025 (78.42)	
	Viral vector-based	22185 (6.64)	55500 (10.83)	
	Recombinant protein	2083 (0.62)	1918 (0.37)	

*chi2.

**Fig 1:**
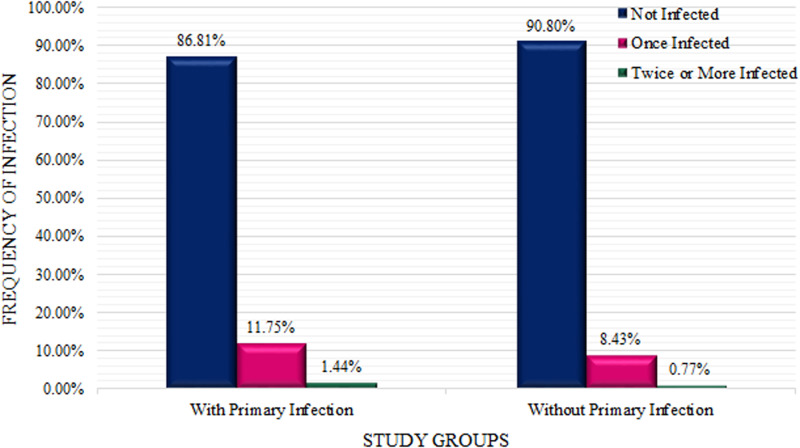
Frequency distribution of characteristics related to primary infection (exposure) and the COVID-19 reinfection in the study population.

In this study, a total of 1,162,128 tests were performed on 846,318 people, and 108,944 COVID-19 reinfections were reported in this population (Fig 1).

In general, 60.54% of the examined people had a positive result in their first PCR test. Regardless of the time interval, only 38.39% of people whose first test was negative (25,674 people) had a positive result in the second test, but this rate reached 71.58% in people whose first test was positive.

Table 2 shows the results of univariate and multivariable analyses using Poisson regression for COVID-19 reinfection in all the populations in the study. The results of this table show that the adjusted rate ratio for primary COVID-19 infection was estimated to be 1.13, which indicates that the incidence rate in people with a history of infection was 13% higher than in people without a history of COVID-19 infection. In addition, the results show that the incidence rate was higher in men, age group under 60 years old, people living in the city, and people who have no history of comorbidity diseases. Also, the results of this analysis indicate that the incidence of COVID-19 reinfections in people who received one dose of the vaccine was 1.33 times higher than in those who did not receive the vaccine, and the incidence rate in people who received two doses of the vaccine was 1.31 times higher than in those who did not receive the vaccine, and this rate reached 1.50 in the group of people who received three or more doses of the vaccine. It should be noted that the incidence rate in people who had received viral vector vaccines was 1.33 times higher than that in people who had not received vaccines. In this analysis, the third group of vaccines, which were recombinant protein vaccines, were excluded from the model due to collinearity (Table 2).

**Table 2:** Univariate and Multivariable analysis using Poisson regression in the study of COVID-19 reinfection in the study population.

Variable		Crude Rate Ratio			Adjusted Rate Ratio		
		Incidence Rate Ratio	P-Value	Confidence Interval	Incidence Rate Ratio	P-Value	Confidence Interval
Primary Infection	Not Infected	Reference	–	–	–	–	–
	Infected	1.24	<0.001	(1.22–1.25)	1.13	<0.001	(1.11-1.14)
Gender	Female	Reference	–	–	–	–	–
	Male	1.26	<0.001	(1.25–1.28)	1.24	<0.001	(1.22-1.25)
Age Group	0–59 years	Reference	–	–	–	–	–
	≥60 years	0.62	<0.001	(0.61–0.63)	0.60	<0.001	(0.59-0.62)
Place of Residence	Rural	Reference	–	–	–	–	–
	City	1.79	<0.001	(1.76–1.83)	1.69	<0.001	(1.66-1.72)
Comorbidities	Without	Reference	–	–	–	–	–
	With	0.73	<0.001	(0.72–0.75)	0.95	<0.001	(0.92-0.97)
Vaccination Status	Unvaccinated	Reference	–	–	–	–	–
	First Dose	1.36	<0.001	(1.32–1.40)	1.33	<0.001	(1.21-1.46)
	Second Doses	1.31	<0.001	(1.28–1.34)	1.31	<0.001	(1.19-1.44)
	Three or More Doses	1.39	<0.001	(1.36–1.42)	1.50	<0.001	(1.36-1.65)
Vaccine Type	Unvaccinated	Reference	–	–	–	–	–
	Inactivated	1.28	<0.001	(1.25–1.31)	0.96	0.480	(0.88-1.06)
	Viral Vector-Based	1.86	<0.001	(1.81–1.91)	1.33	<0.001	(1.21–1.46)
	Recombinant Protein	1.34	<0.001	(1.22–1.47)	Removed due to collinearity		

Table 3 shows the results of univariate and multivariable analyses using Poisson regression for the COVID-19 reinfection in a group of people who received the vaccine before the first PCR test (Table 3).

**Table 3:** Univariate and Multivariable analysis using Poisson regression for the COVID-19 reinfection in first group (people who received vaccine before the first PCR test).

Variable		Crude Rate Ratio			Adjusted Rate Ratio		
		Incidence Rate Ratio	P-Value	Confidence Interval	Incidence Rate Ratio	P-Value	Confidence Interval
Primary Infection	Not Infected	Reference	–	–	–	–	–
	Infected	0.86	<0.001	(0.82–0.89)	0.71	<0.001	(0.68–0.75)
Gender	Female	Reference	–	–	–	–	–
	Male	1.09	<0.001	(1.05–1.14)	1.08	<0.001	(1.03–1.12)
Age Group	0–59 years	Reference	–	–	–	–	–
	≥60 years	0.69	<0.001	(0.66–0.73)	0.69	<0.001	(0.65–0.73)
Place of Residence	Rural	Reference	–	–	–	–	–
	City	2.00	<0.001	(1.86–2.14)	2.02	<0.001	(1.88–2.17)
Comorbidities	Without	Reference	–	–	–	–	–
	With	0.72	<0.001	(0.68–0.77)	0.90	0.006	(0.83–0.97)
Vaccination Status	First Dose	Reference	–	–	–	–	–
	Second Doses	1.27	<0.001	(1.13–1.41)	1.26	<0.001	(1.12–1.40)
	Three or More Doses	1.64	<0.001	(1.48–1.83)	1.79	<0.001	(1.60–1.99)
Vaccine Type	Inactivated	Reference	–	–	–	–	–
	Viral Vector-Based	1.98	<0.001	(1.89–2.07)	1.89	<0.001	(1.81–1.98)
	Recombinant Protein	0.16	0.071	(0.02–1.20)	0.16	0.068	(0.02–1.14)

The results of Table 3 show that in the multivariable analysis, a statistically significant relationship was observed between the primary COVID-19 infection and reinfection, and the incidence rate in people with a history of primary COVID-19 infection was lower than in people without a history of COVID-19 infection (IRR =  0.71). This indicates that primary COVID-19 infection provides a 29% protection against reinfection. In this group, the adjusted rate ratio in men was 1.08 times higher than in women. In addition, a significant relationship between age groups, place of residence, and comorbidities with COVID-19 reinfection was observed. Also, the results of this analysis indicate that the incidence of COVID-19 reinfection in individuals who received two doses of the vaccine was 1.26 times higher than in those who received one dose, and the incidence rate in people who received three doses of vaccine or more was 1.79 times higher than that of those who received a single dose of vaccine. Also, the incidence of COVID-19 reinfection in people who received viral vector vaccines was 1.89 times higher compared to people who received inactivated vaccines.

The analysis was conducted on five classified groups to consider the timing of vaccination in relation to exposure and outcome. Further details about the results of five classified groups can be found in S1-S4 Tables.

## Discussion

In this study, the outcome variable was COVID-19 reinfection, the exposure variable was COVID-19 infection, and other factors were classified as independent or confounding variables. After conducting multivariable regression analysis and adjusting for other variables in both groups, all study variables remained significant in the model.

After adjusting for other variables, the analysis showed that individuals with a positive first PCR test had a rate ratio 1.13 times higher than those with a negative test. Additionally, the adjusted rate ratio for primary COVID-19 infection among individuals vaccinated before their first PCR test was 0.71. These results were in contrast to the study by Sabetian et al., which was conducted on people working in hospitals. The adjusted rate ratio was estimated to be 0.052 in people whose first test was positive compared to people whose first test was negative, and protection against reinfection after previous infection was estimated to be 94.8% [11]. In addition, research conducted in Bangladesh found that the risk of reinfection was significantly lower in individuals with a history of previous infection compared to those without [12]. The results of these studies are not consistent with the results of the present study. A key difference is that previous studies calculated the rate ratio without accounting for vaccine effects, whereas this study includes the vaccine’s impact on infection rates. In this study, a history of COVID-19 infection is considered a risk factor, likely due to its dual impact on susceptibility and immunity. The primary infection can demonstrate the susceptibility of the affected person, and it can also create immunity against subsequent infections. Therefore, it can be concluded that in this research, people were susceptible to the disease to such an extent that this infection could not generate sufficient immunity against subsequent infections.

The results of this study indicate that COVID-19 reinfections were more common in men than in women. A study by Mensah et al. in England estimated the adjusted odds ratio for reinfection to be 0.58. In this study, women had a higher chance of being re-infected with COVID-19 [13]. Also, a meta-analysis study showed that the prevalence of reinfection was higher in men than women [14]. Reviewing the results of different studies shows that the prevalence of reinfection based on gender can be different according to the characteristics of the studied population. According to the results of this study, it can be said that the significant difference observed in the incidence of COVID-19 reinfection in men can be due to the difference in their lifestyles. For instance, men have a significantly higher smoking rate than women. The results of the meta-analysis study showed that smokers are 1.4 times more likely to experience severe symptoms of COVID-19 [15]. In addition, women are more likely to comply with the health guidelines than men [16].

In a study conducted in Serbia by Medic et al., it was shown that people who experienced reinfection with COVID-19 were significantly younger [17]. A multicenter study conducted in Iran also found that the reinfection rate was higher among individuals aged 19 to 64 compared to other age groups [18]. The results of the mentioned study were consistent with the results of the present study. In this study, individuals aged 60 and older had a lower rate ratio than younger participants. This issue in Iran may be due to older people being less present in society because of reverse quarantine, resulting in fewer tests compared to others. Consequently, they may be at lower risk than young people and have a lower incidence of disease. In this regard, a study identified the factors contributing to the higher incidence in younger age groups as follows: younger individuals exhibit a higher cumulative incidence of first infections and face an increased risk of exposure due to social activities. Lower vaccination coverage among young people, who became eligible to receive the vaccine later than older individuals, is another contributing factor. Additionally, this situation may lead to survival bias, where younger individuals survive initial infections due to their lower severity and are therefore more likely to be reinfected [19].

The results of the present study indicate that city residents were significantly more impacted by the COVID-19 reinfection than residents of rural areas. In this regard, a descriptive study in Bangladesh found that city dwellers are more likely to experience COVID-19 reinfection than rural residents [20]. It can be said that the probability of disease transmission in urban areas is higher due to factors such as high population density, industrialization, crowded work environments, and use of public transportation. In addition, city residents are more likely to be tested for COVID-19 than rural residents due to greater awareness and easier access to laboratory services.

In the study “COVID-19 reinfection among infected and vaccinated people”, it was found that at least one comorbidity was present in 50% of people with reinfection [12]. In contrast, research by J. Bean et al. found that comorbidities were not associated with reinfection. Instead, socio-environmental factors were found to be associated with reinfection, rather than comorbidities or immunodeficiency [21]. Another study indicated that the risk of outpatient reinfection was lower among individuals with comorbidities compared to those without [18]. In this regard, the results of the present study indicate that individuals with comorbidities have a lower rate of COVID-19 reinfection compared to those without comorbidities. This could be due to the fact that people with comorbidities often take more precautions to avoid exposure to COVID-19, such as strictly adhering to social distancing, wearing masks, and avoiding crowded places. This heightened awareness and behavior can lead to lower infection rates among these populations. In addition, these individuals frequently interact with healthcare systems because of their illnesses. This contact may facilitate earlier recognition of COVID-19 symptoms, leading to prompt isolation or treatment and ultimately helping to reduce the virus’s spread within this group. Also, individuals with pre-existing conditions often take medication to manage their health, which may also be effective in preventing COVID-19 infection. Another reason for this is that some comorbidities might alter immune responses in ways that could affect susceptibility to infection [22]. For example, people with autoimmune diseases or those on immunosuppressive therapies may have different interactions with the virus compared to healthy individuals. This variability can explain the differing rates of reinfection.

The results of this study indicate that in both groups, vaccination could be considered as a risk factor for contracting a COVID-19 reinfection. In contrast, Cavanaugh’s study reported that unvaccinated individuals had a 2.34 times higher odds of reinfection compared to those who were fully vaccinated [23]. Research by Gazit et al. found that individuals who were both infected and vaccinated were 5.96 times more likely to contract COVID-19 than those who had only been infected [24]. In support of these results, a study conducted in Qatar found that vaccine efficacy decreased significantly six months after the booster dose. This suggests a potential for negative immune imprinting, where individuals who received the booster experienced higher rates of infection compared to those who only completed the primary series of vaccinations [25]. Various studies have reported that the effectiveness of vaccines decreased during the Omicron epidemic compared to previous periods [26–28]. Since the present study was conducted during the spread of this variant, the reasons stated could explain the decrease in vaccine effectiveness. In addition, receiving the vaccine has the greatest effect in reducing hospitalization and death due to COVID-19 and was not effective in reducing cases of infection [29].

In this research, the adjusted rate ratio for those who received viral vector vaccines was estimated to be 1.31 in the entire population and 1.89 in the first group. A study conducted in Costa Rica found that two doses of Pfizer and AstraZeneca vaccines had 93% effectiveness against hospitalization, and one dose had 76% effectiveness [30]. Another study showed that primary vaccination with inactivated vaccines reduced the risk of severe COVID-19 by 74% [31]. These results differ from the present study’s findings that viral vector vaccines are a risk factor for getting a COVID-19 reinfection. It’s possible that viral vector vaccines are more effective against hospitalization and death than infection. Moreover, this study did not consider disease severity unlike previous studies.

## Study limitation

This study has several limitations. First, there is an undercounting of cases because infected individuals who are asymptomatic or have mild symptoms may not seek testing, leading to an underreporting of infections. Additionally, infected individuals with clinical symptoms may also contribute to the undercount if their test results are falsely negative. Second, other factors such as occupational status, body mass index, antibody titer, virus variant, level of self-care, and mask use were not considered in the regression analysis.

## Conclusion

The study reveals that prior COVID-19 infection does not guarantee immunity and may increase the risk of reinfection, particularly among men and younger individuals. It indicates that vaccination complicates this dynamic, as those with multiple doses showed higher reinfection rates than those with fewer doses. The analysis highlights the need for ongoing research to understand the effectiveness of different vaccine types against reinfection. Additionally, it underscores the importance of continuous monitoring of reinfection trends and vaccination strategies. Public health policies must adapt to these findings to effectively address the challenges posed by COVID-19.

## Supporting information

S1 Table Univariate and Multivariable analysis using Poisson regression for the COVID-19 reinfection in second group (unvaccinated people).
(DOCX)

S2 Table Uunivariate and Multivariable analysis using Poisson regression for the COVID-19 reinfection in third group (receiving the vaccine between the first PCR test and the first reinfection).
(DOCX)

S3 Table Univariate and Multivariable analysis using Poisson regression for the COVID-19 reinfection in fourth group (receiving the vaccine between the first and last reinfection).
(DOCX)

S4 Table Univariate and Multivariable analysis using Poisson regression for the COVID-19 reinfection in fifth group (receiving the vaccine after the last reinfection).
(DOCX)
