# Supplementary material for: Role of COVID-19 infection status on the prediction of future infection: Immunity or susceptibility
Source: PLoS One. 2025 Mar 26;20(3):e0317959. doi: 10.1371/journal.pone.0317959 (PMC11940750; doi:10.1371/journal.pone.0317959)
Supplement: S1 Table — (DOCX) [file pone.0317959.s001.docx]

S1 Table. Univariate and Multivariable analysis using Poisson regression for the new Covid-19 infection in second group (unvaccinated people)

| Variable | | Crude Rate Ratio | | | Adjusted Rate Ratio | | |
| --- | --- | --- | --- | --- | --- | --- | --- |
|  |  | Incidence Rate Ratio | P-Value | Confidence Interval | Incidence Rate Ratio | P-Value | Confidence Interval |
| Primary Infection | Not Infected | Reference | - | - | - | - | - |
|  | Infected | 2.03 | <0.001 | (1.94-2.13) | 1.93 | <0.001 | (1.85-2.03) |
| Gender | Female | Reference | - | - | - | - | - |
|  | Male | 1.14 | <0.001 | (1.09-1.18) | 1.15 | <0.001 | (1.10-1.19) |
| Age Group | 0-59 Years | Reference | - | - | - | - | - |
|  | ≥60 Years | 0.74 | <0.001 | (0.67-0.81) | 0.69 | <0.001 | (0.63-0.75) |
| Place of Residence | Rural | Reference | - | - | - | - | - |
|  | City | 2.22 | <0.001 | (2.05-2.41) | 1.91 | <0.001 | (1.76-2.08) |
| Comorbidities | Without | Reference | - | - | - | - | - |
|  | With | 0.98 | 0.738 | (0.89-1.08) | - | - | - |
