# Supplementary material for: Role of COVID-19 infection status on the prediction of future infection: Immunity or susceptibility
Source: PLoS One. 2025 Mar 26;20(3):e0317959. doi: 10.1371/journal.pone.0317959 (PMC11940750; doi:10.1371/journal.pone.0317959)
Supplement: S2 Table — (DOCX) [file pone.0317959.s002.docx]

S2 Table. Univariate and Multivariable analysis using Poisson regression for the new Covid-19 infection in third group (receiving the vaccine between the first PCR test and the first new infection)

| Variable | | Crude Rate Ratio | | | Adjusted Rate Ratio | | |
| --- | --- | --- | --- | --- | --- | --- | --- |
|  |  | Incidence Rate Ratio | P-Value | Confidence Interval | Incidence Rate Ratio | P-Value | Confidence Interval |
| Primary Infection | Not Infected | Reference | - | - | - | - | - |
|  | Infected | 1.38 | <0.001 | (1.35-1.41) | 1.21 | <0.001 | (1.19-1.24) |
| Gender | Female | Reference | - | - | - | - | - |
|  | Male | 1.26 | <0.001 | (1.24-1.28) | 1.22 | <0.001 | (1.20-1.25) |
| Age Group | 0-59 Years | Reference | - | - | - | - | - |
|  | ≥60 Years | 0.88 | <0.001 | (0.86-0.91) | 0.81 | <0.001 | (0.79-0.84) |
| Place of Residence | Rural | Reference | - | - | - | - | - |
|  | City | 1.93 | <0.001 | (1.88-1.99) | 1.76 | <0.001 | (1.71-1.82) |
| Comorbidities | Without | Reference | - | - | - | - | - |
|  | With | 0.83 | <0.001 | (0.81-0.86) | 0.92 | <0.001 | (0.89-0.95) |
| Vaccination Status | First Dose | Reference | - | - | - | - | - |
|  | Second Doses | 1.34 | <0.001 | (1.29-1.39) | 1.36 | <0.001 | (1.31-1.41) |
|  | Three or More Doses | 1.72 | <0.001 | (1.66-1.78) | 1.82 | <0.001 | (1.76-1.89) |
| Vaccine Type | Inactivated | Reference | - | - | - | - | - |
|  | Viral Vector-Based | 2.02 | <0.001 | (1.98-2.07) | 1.92 | <0.001 | (1.88-1.97) |
|  | Recombinant Protein | 0.28 | <0.001 | (0.22-0.36) | 0.35 | <0.001 | (0.27-0.45) |
