# Supplementary material for: Role of COVID-19 infection status on the prediction of future infection: Immunity or susceptibility
Source: PLoS One. 2025 Mar 26;20(3):e0317959. doi: 10.1371/journal.pone.0317959 (PMC11940750; doi:10.1371/journal.pone.0317959)
Supplement: S3 Table — (DOCX) [file pone.0317959.s003.docx]

S3 Table. Univariate and Multivariable analysis using Poisson regression for the new Covid-19 infection in fourth group (receiving the vaccine between the first and last new infection)

| Variable | | Crude Rate Ratio | | | Adjusted Rate Ratio | | |
| --- | --- | --- | --- | --- | --- | --- | --- |
|  |  | Incidence Rate Ratio | P-Value | Confidence Interval | Incidence Rate Ratio | P-Value | Confidence Interval |
| Primary Infection | Not Infected | Reference | - | - | - | - | - |
|  | Infected | 0.87 | <0.001 | (0.83-0.90) | 0.86 | <0.001 | (0.83-0.90) |
| Gender | Female | Reference | - | - | - | - | - |
|  | Male | 0.99 | 0.916 | (0.95-1.03) | 0.99 | 0.936 | (0.95-1.03) |
| Age Group | 0-59 Years | Reference | - | - | - | - | - |
|  | ≥60 Years | 0.92 | 0.099 | (0.85-1.01) | 0.94 | 0.202 | (0.86-1.03) |
| Place of Residence | Rural | Reference | - | - | - | - | - |
|  | City | 0.99 | 0.886 | (0.93-1.06) | - | - | - |
| Comorbidities | Without | Reference | - | - | - | - | - |
|  | With | 0.96 | 0.436 | (0.89-1.04) |  | - | - |
| Vaccination Status | First Dose | Reference | - | - | - | - | - |
|  | Second Doses | 0.97 | 0.408 | (0.91-1.03) | 0.96 | 0.362 | (0.90-1.03) |
|  | Three or More Doses | 0.93 | 0.054 | (0.87-1.00) | 0.93 | 0.036 | (0.87-0.99) |
| Vaccine Type | Inactivated | Reference | - | - | - | - | - |
|  | Viral Vector-Based | 0.98 | 0.667 | (0.93-1.04) | 0.98 | 0.530 | (0.93-1.03) |
|  | Recombinant Protein | 1.13 | 0.496 | (0.79-1.62) | 1.08 | 0.667 | (0.75-1.55) |
