# Supplementary material for: Role of COVID-19 infection status on the prediction of future infection: Immunity or susceptibility
Source: PLoS One. 2025 Mar 26;20(3):e0317959. doi: 10.1371/journal.pone.0317959 (PMC11940750; doi:10.1371/journal.pone.0317959)
Supplement: S4 Table — (DOCX) [file pone.0317959.s004.docx]

S4 Table. Univariate and Multivariable analysis using Poisson regression for the new Covid-19 infection in fifth group (receiving the vaccine after the last new infection)

| Variable | | Crude Rate Ratio | | | Adjusted Rate Ratio | | |
| --- | --- | --- | --- | --- | --- | --- | --- |
|  |  | Incidence Rate Ratio | P-Value | Confidence Interval | Incidence Rate Ratio | P-Value | Confidence Interval |
| Primary Infection | Not Infected | Reference | - | - | - | - | - |
|  | Infected | 0.83 | <0.001 | (0.81-0.85) | 0.83 | <0.001 | (0.81-0.85) |
| Gender | Female | Reference | - | - | - | - | - |
|  | Male | 0.98 | 0.188 | (0.96-1.00) | 0.97 | 0.075 | (0.95-1.00) |
| Age Group | 0-59 Years | Reference | - | - | - | - | - |
|  | ≥60 Years | 0.93 | 0.009 | (0.89-0.98) | 0.94 | 0.028 | (0.89-0.99) |
| Place of Residence | Rural | Reference | - | - | - | - | - |
|  | City | 1.01 | 0.548 | (0.97-1.04) | - | - | - |
| Comorbidities | Without | Reference | - | - | - | - | - |
|  | With | 0.95 | 0.048 | (0.91-0.99) | 0.96 | 0.113 | (0.91-1.00) |
